# 946. Establishing Antimicrobial Stewardship Programme at Four Secondary Care Hospitals in India through Hub and Spoke Model with Christian Medical College, Vellore as Guiding Centre

**DOI:** 10.1093/ofid/ofac492.789

**Published:** 2022-12-15

**Authors:** Prasanna Kumar, Priscilla Rupali

**Affiliations:** CHRISTIAN MEDICAL COLLEGE, VELLLORE, Tamil Nadu, India; Christian Medical College, Vellore, Tamil Nadu, India

## Abstract

**Background:**

The high burden of antimicrobial resistance in India necessitates an urgent implementation of Antimicrobial Stewardship programs (ASP). Most ASP are based at tertiary care centres, with sparse data available regarding the effectiveness of an ASP in a low resource primary/secondary care setting.

**Methods:**

We adopted a hub and spoke model to implement ASP in these low resource settings. The study consisted of three phases. Initial phase captured baseline antimicrobial days of therapy DOTs data with no feedback, followed by intervention phase wherein a few chosen interventions were implemented. Physicians of four chosen hospitals were trained via a blended customized distance education training programme, assisted with development of antibiogram based on their local hospital microbial resistance patterns via WHONET, followed by development of hospital specific antibiotic policy and augmentation of the existing laboratory skills by training personnel at the central facility. This was followed by a post intervention phase with a prospective review and feedback by trained personnel with assessment of DOTs.

**Results:**

During the baseline phase, 1459 patients from all four sites were enrolled; 1233 patients were enrolled in the intervention phase. Both groups had comparable baseline characteristics. The key outcome, DOT per 1,000 patient days, was 1952.63 in the baseline phase. The DOT/1000 patient days was significantly lower in the post intervention period, at 1483.06 (P =0.001). Quinolones, Macrolides, Cephalosporins, Clindamycin, and Nitroimidazole use significantly decreased in the post intervention phase. Rate of de-escalation was significantly higher in the intervention phase compared to the baseline phase (44% vs 12.5%; P < .0001), suggesting a definite trend with regard to the judicious use of antibiotics. Post intervention phase revealed that 79.9% of antibiotic use was justified. Overall, the recommendations given by the ASP team were fully followed in 946 cases (77.7%), partially followed in 59 cases (4.8%), and not followed in 137 cases (35.7%). No adverse events were noted.

**Conclusion:**

Our Hub and Spoke model of ASP was successful in implementing ASP in secondary care hospitals in India which is the need of the hour.

**Disclosures:**

**Priscilla Rupali, MD, DTM&H**, PFIZER: Grant/Research Support|PFIZER: Grant/Research Support.

